# Pregnancy and COVID-19: The Possible Contribution of Vitamin D

**DOI:** 10.3390/nu14163275

**Published:** 2022-08-10

**Authors:** Alessandra Manca, Stefano Cosma, Alice Palermiti, Martina Costanzo, Miriam Antonucci, Elisa Delia De Vivo, Alice Ianniello, Fulvio Borella, Andrea Roberto Carosso, Silvia Corcione, Francesco Giuseppe De Rosa, Chiara Benedetto, Antonio D’Avolio, Jessica Cusato

**Affiliations:** 1 Laboratory of Clinical Pharmacology and Pharmacogenetics, Department of Medical Sciences, University of Turin, Amedeo di Savoia Hospital, 10149 Turin, Italy; 2 Obstetrics and Gynecology Unit 1, Department of Surgical Sciences, Sant’Anna Hospital, University of Turin, 10126 Turin, Italy; 3 ASL Città di Torino, Amedeo di Savoia Hospital, 10149 Turin, Italy; 4 Unit of Infectious Diseases, Department of Medical Sciences, University of Turin, Città della Salute e della Scienza, 10126 Turin, Italy

**Keywords:** SARS-CoV-2, vitamin D, genetic polymorphisms, *VDR*, biomarkers, newborn

## Abstract

Background: Vitamin D deficiency has been associated with the severity of COVID-19. The role of vitamin D in pregnant women with COVID-19 has been poorly investigated to date. The aim of this study was to evaluate the influence of vitamin D in affecting some clinical features in pregnancy between SARS-CoV-2 positive and negative patients. Methods: Vitamin D pathway related polymorphisms and 25-hydroxyvitamin D levels were quantified in pregnant women followed from the first to the third trimester of pregnancy. Vitamin D deficiency was considered with values ≤ 30 ng/mL. Results: In total, 160 women were enrolled: 23 resulted positive for at least one SARS-CoV-2 related test (molecular swab or antibody tests). Vitamin D-associated polymorphisms were able to affect vitamin D levels in SARS-CoV-2 negative and positive subjects: remarkably, all the *VDR* TaqICC genotype patients were negative for SARS-CoV-2. In a sub-population (118 patients), vitamin D levels correlated with pregnancy-related factors, such as alpha-fetoprotein levels. Third-trimester vitamin D levels were lower in preterm births compared to full-term pregnancy: this trend was highlighted for SARS-CoV-2 positive patients. Conclusions: This is the first study demonstrating a role of vitamin D in affecting the clinical characteristics of pregnant women during the COVID-19 era. Further studies in larger and different cohorts of patients are required to confirm these findings.

## 1. Introduction

In the last few years, coronaviruses (CoVs) have caused infections around the world, especially affecting the respiratory system. In particular, the “Severe Acute Respiratory Syndrome” (SARS) from SARS-CoV in China in 2002 and the “Middle East Respiratory Syndrome” (MERS) from MERS-CoV in 2012 in Saudi Arabia were the most severe. A new CoV (SARS-CoV-2) developed in China in 2019 and was officially declared a pandemic by World Health Organization on 11 March 2020 [1]. The disease, called COVID-19, leads to severe flu-like and gastrointestinal symptoms, including fever, headache, cough, dyspnea and, in the long term, myocarditis.

In some cases, a “cytokine storm” can occur, leading to pneumonia, respiratory failure (acute respiratory distress syndrome (ARDS)) and renal failure, eventually leading to death [2]. Altered acquired immune responses and uncontrolled innate inflammatory responses to SARS-CoV-2 can cause cytokine storms, which exaggerate the pathology together with the progression of thrombosis, and disseminated intravascular coagulation [3]. The most frequently reported coagulation abnormality in COVID-19 is the increase in D-dimer and thrombosis, including macro- and micro-thrombosis [4].

It is known that immune response, inflammation and coagulation are modulated by vitamin D activity [5,6]. In particular, vitamin D induces the conversion of monocytes to macrophages and influences the activity of dendritic, T and B cells. It also enhances cellular immunity by reducing the cytokine storm induced by the innate immune system, which generates both pro-inflammatory and anti-inflammatory cytokines in response to viral infections, as observed in COVID-19 patients [7].

In addition, vitamin D can alter the development of inflammatory T helper 17 (Th17) cell type mass towards anti-inflammatory regulatory T cell populations (T-reg cells), reducing the levels of pro-inflammatory cytokines such as IL-1, IL-6 and TNF-α, simultaneously increasing the level of anti-inflammatory IL-10 [8,9].

Vitamin D metabolites have an effective anticoagulant activity regulating several pro- and anti-thrombotic agents of the coagulation cascade. In particular, they can upregulate the expression of the endogenous anticoagulant thrombomodulin and downregulate the expression of the tissue factor, a crucial pro-thrombotic element involved in the early steps of the coagulation activation [5].

The role of vitamin D in pregnant women with COVID-19 has been poorly investigated to date. During pregnancy, vitamin D alters anti-Müllerian hormone signaling, follicle-stimulating hormone sensitivity, and progesterone production and release in human granulosa cells, indicating a possible physiologic role for vitamin D in ovarian follicular development and luteinization. In detail, 25-hydroxyvitamin D is positively correlated with anti-Müllerian hormone concentrations: consequently, vitamin D supplementation can reduce the seasonal changes in anti-Müllerian levels [10].

Schmitt et al. have recently measured vitamin D levels and attempted to correlate them with the severity of COVID-19 [11]. They found that both positive and negative women had low levels of vitamin D in the third trimester. However, the deficiency was greater in women with mild COVID-19. To date, no data are available concerning the role of vitamin D in affecting SARS-CoV-2 test positivity in the different pregnancy periods and some clinical features.

For this reason, the aim of this study was to quantify 25-hydroxyvitamin D during the three trimesters; in addition, polymorphisms of genes related to the vitamin D pathway were analyzed in order to understand their role in affecting positivity and some clinical features, comparing healthy subjects with patients affected by COVID-19.

## 2. Materials and Methods

### 2.1. Patient Enrolment

Pregnant women were enrolled from April to December 2020 at the Sant’Anna Hospital in Turin. Biological samples were obtained during the three different trimesters of pregnancy: For the first sample collection, a test tube containing EDTA for genetic analysis, a red test tube without anticoagulant for serological tests for SARS-CoV-2 and for 25-hydroxyvitamin D quantification, and the molecular swab were collected. Only swabs and red test tubes were drawn during the second- and third-trimester visits. Patients were divided into two groups: positive for at least one test for SARS-CoV-2 (molecular swabs or serological tests) and negative individuals. The study was conducted after approval by the Ethics Committee (No. 00171/2020).

### 2.2. SARS-CoV-2 Detection Methods

#### 2.2.1. Molecular Swabs

Viral RNA was extracted using the MagNA Pure compact instrument (Roche, Basel, Switzerland) and it was analyzed using a real-time polymerase chain reaction system (RT-PCR) (CFX-96, Bio-Rad, Hercules, CA, USA) with the Liferiver Novel Coronavirus 2019-nCoV Real-Time RT-PCR kit protocol for the N, E and ORF1ab genes (Liferiver Bio-Tech, San Diego, CA, USA). Its negative percentage agreement was 100%.

#### 2.2.2. Detection of Antibodies against SARS-CoV-2

Different methods for antibody evaluation were used. A rapid lateral flow immunochromatographic device (PRIMALAB, Balerna, Switzerland) was applied for the qualitative detection of immunoglobulin (Ig) M and IgG; its sensitivity was 98.92% (95% CI: 94.2–99.8%) for IgG and 92.98% (95% CI: 83.3–97.2%) for IgM compared to the reference method (ELISA). The combined IgG and IgM specificity was 98.3% (95% CI: 95.2–99.4%). A CE-approved automated rapid fluorescent lateral flow immunoassay (AFIAS COVID-19, Boditech, Gang-won-do, Korea) was employed for the semi-quantitative determination of non-neutralizing IgM and IgG antibodies to viral proteins S and N. Semi-quantitative results were expressed as cut-off index (COI), and a COI > 1.1 indicates a positive result. The positive percentage agreement was 95.8% and the negative percentage agreement was 96.7%.

Finally, another CE-approved chemiluminescent immunoassay technology (Liaison SARS-CoV-2 S1/S2 IgG, DiaSorin, Saluggia, Italy) was used as a reference test for the semi-quantitative detection of specific neutralizing antibodies only of the anti-S1 and anti-S2 IgG types against SARS-CoV-2. Antibody concentration was expressed as an arbitrary unit (AU/mL), and results are classified as positive when ≥15 AU/mL. The positive percentage agreement was 94.4% and the negative percentage agreement was 97.8%.

### 2.3. Genetic Polymorphism Analyses

The “QIAamp DNA mini kit” (Qiagen, Valencia, CA, USA) was used for genomic DNA extraction. These kits contain columns allowing DNA purification starting from 200 µL of blood or plasma.

Allelic discrimination was assessed using RT-PCR (BIORAD, Milan, Italy).

The following allelic variants were analyzed: 

*VDR* ApaI C > A (rs 7975232), *VDR* TaqI T > C (rs 731236), *VDR* BsmI G > A (rs 1544410), *VDR* FokI T > C (rs 17535810), *VDR* Cdx2 A > G (rs 11568820), *VDBP* GC1296 A > C (rs 7041), *CYP27B1* −1260 G > T (rs 10877012), *CYP24A1* 3999 T > C (rs 2248359), *CYP24A1* 8620 A > G (rs 2585428), *CYP27B1* +2838 C > T (rs 4646536), *CYP27A1* 345 A > G (rs 4674345) and *CYP24A1* 22776 C > T (rs 927650).

### 2.4. 25-Hydroxyvitamin D Quantification

The MSMS vitamin D kit (Perkin Elmer, Wallac Oyster, Finland) was used for the quantitative determination of 25-vitamin D from human plasma samples, which were analyzed by liquid chromatography coupled with tandem mass spectrometry (LC-MS/MS). The method’s imprecision and inaccuracy were lower than 10%, confirmed during each analytical run by internal quality controls. Quantifications were carried out in the first (25-vitamin D1), second (25-vitamin D2) and third (25-vitamin D3) trimester. Vitamin D deficiency was considered for values ≤ 30 ng/mL.

### 2.5. Statistical Analysis

The Shapiro–Wilk test was used to test the normality in the data distribution. Non-normally distributed continuous variables were given as median and interquartile range (IQR), normally distributed continuous variables were given as mean ± standard deviation, and categorical variables were described as numbers and percentages. Kruskal–Wallis and Mann–Whitney tests were adopted to test differences for continuous variables and genetic groups, considering the level of statistical significance (*p*-value) < 0.05.

Correlations were evaluated through Spearman tests (Spearman coefficient (SC)). The predictive power of the considered variables was finally evaluated through univariate and multivariate logistic regression analysis (*p*-value = *p*; odd ratio = OR; interval of confidence = IC 95%).

All the tests were performed with IBM SPSS Statistics 27.0 for Windows (Chicago, IL, USA).

## 3. Results

### 3.1. Patients’ Characteristics

In this study, 160 pregnant women were enrolled and followed up from the first to the third trimester of pregnancy: 15 (9.4%) tested positive for the molecular swabs identifying SARS-CoV-2, and 8 (5%) tested positive only for serological tests detecting anti-SARS-CoV-2 antibodies; accordingly, 23 (14.4%) were considered positive for both the analyses (molecular swabs and serological tests). The main clinical features of the pregnant women enrolled in the present study are summarized in Table 1. 

### 3.2. Vitamin D Polymorphisms, Vitamin D Levels and COVID-19

The allele frequencies of the genetic variants are shown in Supplementary Table S1: the Hardy–Weinberg equilibrium was demonstrated for all polymorphisms, with the exception of *VDR* BsmI, *VDR* FokI, *VDR* Cdx2 and *CYP27B1* −1260.

The role of vitamin D single nucleotide polymorphisms (SNPs) in affecting this pro-hormone levels was evaluated: for negative individuals, *CYP24A1* 8620 GG was associated with 25-vitamin D2 (*p* = 0.03), whereas *CYP27A1* 345 AG/GG was associated with 25-vitamin D1 (*p* = 0.03), 25-vitamin D2 (*p* = 0.04) and 25-vitamin D3 (*p* = 0.002); for positive patients, *CYP24A1* 3999 CC was related to 25-vitamin D2 (*p* = 0.05) and 25-vitamin D3 (*p* = 0.04). No differences in vitamin D levels between negative and positive patients for at least one test in different trimesters were suggested.

Furthermore, the impact of genetic polymorphisms on positivity was assessed: only the CC genotype for the *VDR* TaqI polymorphism was statistically significant (*p* = 0.04).

**Figure 1:**
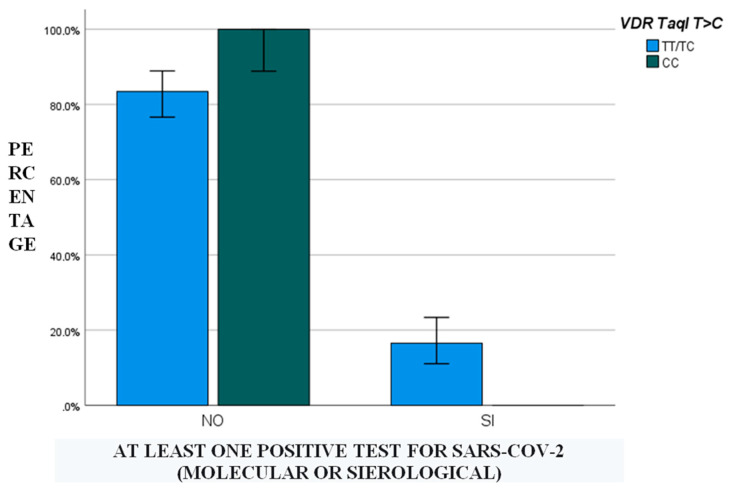
Graph showing that none of the patients with the CC genotype for the *VDR* TaqI polymorphism (CC) ever tested positive for SARS-CoV-2.

Finally, a binary logistic regression analysis was performed in order to assess which demographic, genetic, pharmacological, clinical and/or hematochemical factors were able to predict the positivity of at least one test: in the univariate regression model, vitamin D levels < 10 ng/mL in the first and third trimesters of pregnancy (*p* < 0.001 for both), administration of low-molecular-weight heparin (*p* = 0.04) and CC genotype for the *VDR* TaqI polymorphism (*p* < 0.001) were retained. In the multivariate regression, *VDR* TaqI polymorphism was the only predictor (*p* < 0.001): in detail, all CC patients showed negativity for every SARS-CoV-2-related test (Figure 1).

**Figure 2:**
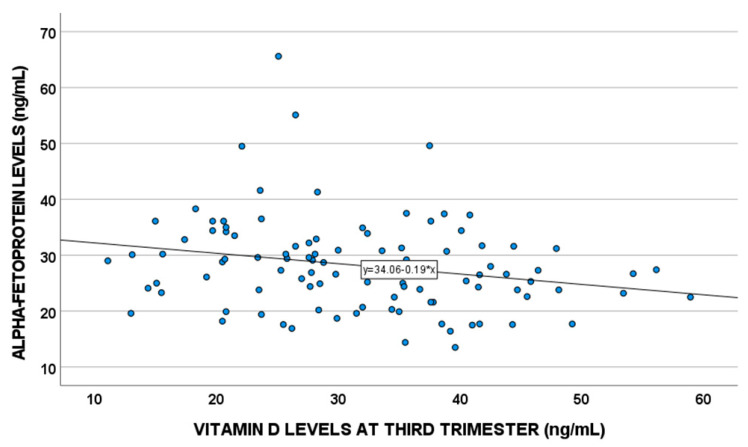
Vitamin D levels and alpha-fetoprotein correlation (*p* = 0.02, SC = −0.24).

In a sub-population of 118 patients, vitamin D levels were correlated with some pregnancy-related factors, such as alpha-fetoprotein (AFP) levels (*p* = 0.02, SC = −0.24) (Figure 2).

**Figure 3:**
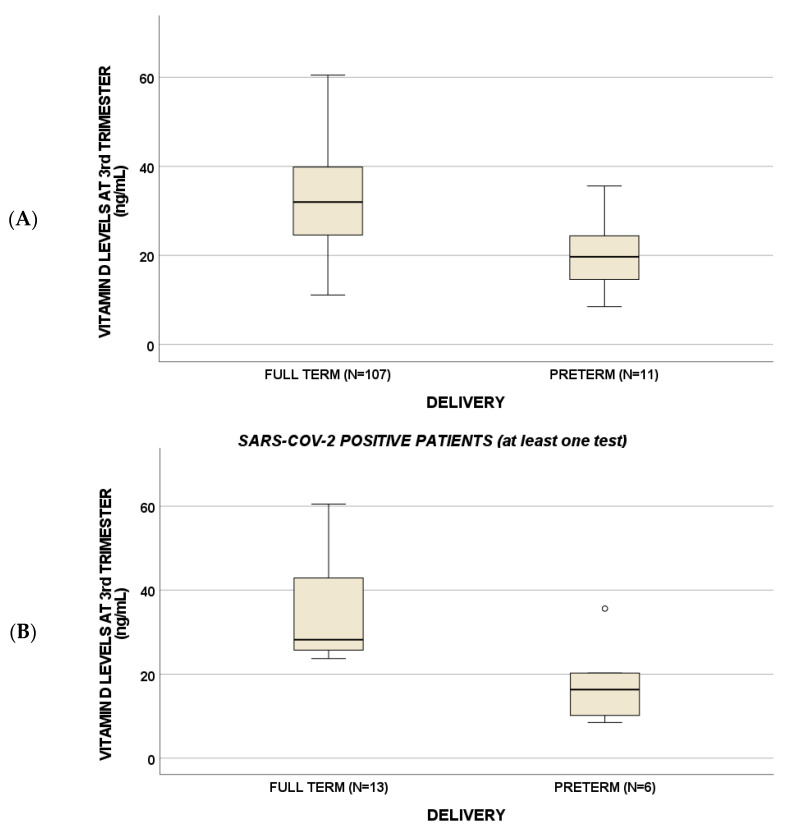

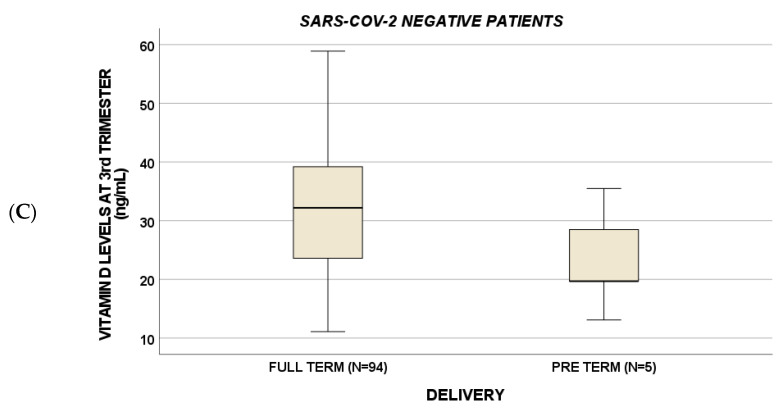
Third-trimester vitamin D levels were lower in preterm births compared to full-term pregnancy (*p* < 0.001) (**A**): in particular, a *p*-value of 0.01 was suggested for positive subjects (**B**), whereas *p* = 0.07 for negative women (**C**). Circles indicate "out" values.

Different vitamin D pathway related SNPs were able to affect some clinical features (Table 2): in particular, *VDR* ApaI AA genotype was associated with preterm birth (PTB) in positive patients (*p* = 0.03). Moreover, third-trimester vitamin D levels were lower in PTB compared to full-term pregnancy (*p* < 0.001, Table 3, Figure 3a): in particular, this trend was evidenced for both SARS-CoV-2 positive and negative patients (at least one test), but a strong *p*-value of 0.01 was suggested for positive subjects (Figure 3b), whereas a borderline statistical significance (*p* = 0.07) was found for SARS-CoV-2 negative women (Figure 3c).

Finally, a binary logistic analysis considering all demographic, pharmacological and vitamin D-associated factors predicting PTB was performed: at least one positivity for SARS-CoV-2 tests, 25-vitamin D1 ≤ 30 ng/mL, 25-vitamin D2 ≤ 30 ng/mL, 25-vitamin D3 ≤ 30 ng/mL, *VDR* TaqI CC, *VDR* FokI TC/CC, *VDR* Cdx2 GG, *CYP27B1* −1260 TT and *CYP27B1* +2838 CT/TT remained in the univariate model; no factors were retained in the multivariate model.

## 4. Discussion

COVID-19 has an impact on pregnancy and children: As an example, maternal SARS-CoV-2 positivity during pregnancy was related to a higher rate of neurodevelopmental sequelae in unadjusted models compared to those a model adjusted for race, ethnicity, insurance status, offspring sex, maternal age and preterm status. In addition, third-trimester infection was associated with effects of a larger magnitude [12]. In particular, SARS-CoV-2 leads to unique inflammatory responses at the maternal–fetal interface and induces humoral and cellular immune responses in the maternal blood, as well as mild cytokine production in the neonatal circulation [13].

Vitamin D is structurally related to classic steroid hormones. It is synthesized from cholecalciferol in the skin through a chemical reaction depending on sunlight exposure. Then, it is transported by the vitamin D binding protein (VDBP) to the liver, where it is hydroxylated at carbon 25, resulting in 25-vitamin D, the main circulating form [6,13]. In the kidney, this molecule is converted into 1,25-vitamin D or calcitriol (the most active metabolite) by the enzyme 1-α-hydroxylase (*CYP27B1*). 1,25-Vitamin D levels are tightly controlled by *CYP24A1*, which converts 25-D and 1,25-vitamin D into 24,25-vitamin D and 1,24,25-vitamin D [14].

Vitamin D exerts its biological functions through its nuclear receptor (VDR), which acts as a ligand-activated transcriptional factor [6,7].

In this study, we documented that vitamin D-associated genetic variants were able to influence vitamin D levels in pregnant women and were significantly associated with the positivity for at least one SARS-CoV-2 test (molecular swab or antibody test).

In literature, a comparative case–control study in a pregnant population of 82 positive and 174 negative women (controls) suggested serum 25-vitamin D levels were significantly lower in positive pregnant women than in controls at the time of delivery [11]. However, as the main study limitation, only a single timing of blood collection was considered. Our study extended previous findings by confirming the predictive role of vitamin D-associated polymorphisms during all three trimesters of pregnancy. 

As additional findings, in a sub-population of patients, we documented that vitamin D levels in the third trimester were lower in PTB compared to full-term pregnancy and were associated with some pregnancy-related factors, such as alpha-fetoprotein (AFP) concentrations.

We found reduced vitamin D levels in the third trimester of pregnancy associated with PTB. These findings could be justified by the inverse correlation suggested for vitamin D and AFP in this work. In fact, higher levels of this protein could be the cause of placental abruption and thus PTB (the role of unexplained high serum AFP and human chorionic gonadotropin levels in the second trimester determining poor obstetric outcomes) [15,16]. Remarkably, these trends were confirmed for all the enrolled women, but they were more statistically significant for COVID-19 patients. These findings were indirectly confirmed by another study, which evaluated 15 SARS-CoV-2 positive pregnant women (7 asymptomatic and 8 mildly symptomatic) and 20 healthy pregnant controls and showed that vitamin D deficiency (<20 ng/mL) was present in all pregnant women but with lower levels (<12 ng/mL) found in pregnant women with mild COVID-19 [17]. Similarly, other studies reported lower 25-vitamin D levels in SARS-CoV-2 positive pregnant women, also suggesting vitamin D supplementation during the gestation [17,18].

Different works suggested vitamin D deficiency during pregnancy could produce an effect on PTB [19,20,21]: A systematic review and meta-analysis of 24 observational studies was performed to clarify this topic. On one hand, a study highlighted vitamin D deficiency during gestation did not increase the risk of PTB. On the other hand, subgroup analysis indicates that vitamin D deficiency in the second trimester may increase PTB risk. Vitamin D levels should be measured in the second trimester of pregnancy, and vitamin D supplements should be recommended [19].

Furthermore, in the multivariate regression analysis suggesting factors able to predict SARS-CoV-2 test positivity, *VDR* TaqI SNP was the only factor retained in the final model: in particular, CC genotype patients compared to CT/TT group always presented negativity for every test. The *VDR* TaqI polymorphism is present in the coding region of exon 9 of the 12q13.11 gene and encodes for VDR, the nuclear receptor for vitamin D [14]. In the literature, the T allele, compared to the C allele, has been found associated with asthma and also with tuberculosis, chronic hepatitis, lepromatous leprosy and type 1 diabetes in Eastern Europeans and South Indians [22]. Our study seems to confirm what was published in the literature: in fact, the CC genotype was found to be protective against SARS-CoV-2 infection.

During pregnancy, physiological and immunological changes normally occur in a woman in order to tolerate the genetically foreign fetus. Thus, pregnant women are more vulnerable to viral infections, which lead to adverse obstetric and neonatal outcomes, such as those observed for COVID-19 [23]. In this context, different papers suggested PTB as the most common adverse neonatal outcome in COVID-19-affected patients, but the mechanisms have not been yet clarified. 

This preliminary study could suggest vitamin D implications in pregnancy processes, other than in COVID-19: it is important to highlight that vitamin D affects the expression and concentration of Angiotensin Converting Enzyme 2 (ACE2), the SARS-CoV-2 receptor, which is largely expressed in the ovary, vagina, uterus and placenta [24,25]. Angiotensin II, ACE2 and angiotensin-(1–7) modulate follicle development and ovulation, control luteal angiogenesis and degeneration and affect endometrial tissue and embryo development. For this reason, SARS-CoV-2 infection could perturb the female reproductive system through its action on ACE2 [24]. In detail, vitamin D modulates and inhibits renin expression. Vitamin D can provoke ACE2/Ang-(1–7)/MasR axis activity and arrest renin and the ACE/Ang II/AT1R axis, increasing the expression and concentration of ACE2, MasR and Ang-(1–7), and consequently plays a protective role in combating acute lung injury/acute respiratory distress syndrome. Vitamin D also contests the virus with different mechanisms: pro-inflammatory cytokine reduction, anti-inflammatory cytokine increase and activation of defensive cells such as macrophages. Vitamin D might alleviate SARS-CoV-2 complications by ACE2 modulation. Indeed, a possible vitamin D therapeutic approach for treating COVID-19 aims at affecting the downregulation of RAS and ACE2, also in addition to angiotensin type 1 receptor (AT1R), which also can increase the expression of ACE2. In the literature, vitamin D supplementation was shown to increase VDR and ACE2 mRNA expression in a murine model of lipopolysaccharide-induced acute lung injury, suggesting a protective role of vitamin D against acute lung injury [25].

Our findings could be important to improve the management of the pregnant population: early pharmacogenetic analyses combined with vitamin D monitoring might in fact allow the identification of patients at risk of pregnancy-related complications that may benefit from personalized vitamin D supplementation. 

One of the strengths of this study is the comparison of the vitamin D levels of SARS-CoV-2 negative and positive pregnant women in three trimesters for the first time. Another strength is that the genetic influence has been considered.

The limits of this study were the small numbers of SARS-CoV-2-affected patients and PTBs; moreover, additional factors associated with inflammation, coagulation or symptoms could be evaluated according to genetics in further analyses. Despite these limitations, we believe that our study provides some important hypothesis-generating findings that deserve further future investigations.

## 5. Conclusions

In conclusion, this is the first study showing a possible role of vitamin D concentrations and genetic polymorphisms in affecting COVID-19 clinical features in pregnant women; however, studies in larger and independent cohorts are needed to confirm these data.

## Figures and Tables

**Table 1:** Patients’ characteristics (IQR = interquartile range).

Characteristics	Value
Number of patients (*n*)	160
Age (years), median (IQR)	32 (28)
Positive molecular swabs, *n* (%)	15 (9)
At least one positive molecular swab or serological test, *n* (%)	23 (14)
Cardioaspirin administration, *n* (%)	4 (3)
Administration of low-molecular-weight heparin, *n* (%)	2 (1)
Amniocentesis, *n* (%)	5 (3)
Malformations, *n* (%)	3 (2)
Abortions, *n* (%)	2 (1)
Preterm birth, *n* (%)	15 (11)
Alpha-fetoprotein (ng/mL), median (IQR)	1 (0.9–1.2)
Estriol (ng/mL), median (IQR)	1 (0.9–1.3)
Human chorionic gonadotropin (mlU/mL), median (IQR)	1 (1.8–1.4)
Pregnancy-associated plasma protein A (UI/L), median (IQR)	1 (0.7–1.6)
Nuchal translucency (mm), median (IQR)	1.1 (0.9–1.2)
Vitamin D levels in first trimester (ng/mL), median (IQR)	23 (17–29)
Vitamin D levels in second trimester (ng/mL), median (IQR)	25 (19–35)
Vitamin D levels in third trimester (ng/mL), median (IQR)	30 (23–39)

**Table 2:** Influences of genetic polymorphisms with pregnancy characteristics in patients negative on every test for SARS-CoV-2 (A) and patients with at least one positive on a test for SARS-CoV-2 (B) (E3 = estriol; AFP = alpha-fetoprotein; NT = nuchal translucency; ABD-C = abdominal circumference; FFL = fetal femur length; MAL = malformations; GD = gestational diabetes; AB = abortion; LRF DNA GD = low risk fetal DNA for genetic disease; AMNIO = amniocentesis; PTB = preterm birth).

	*p*-Values for Patients Negative for Every Test for SARS-CoV-2										
A	E3	AFP	NT	ABD-C	FFL	MAL	GD	AB	LRF DNA GD	AMNIO	PTB
*VDR* Taq I (TC_CC)		0.02								0.003	
*VDR* Taq I (CC)						0.02					
*VDR* ApaI (CA_AA)		0.03									
*VDR* FokI (CC)		0.03									
*VDR* FokI (TC_CC)	0.04										
*VDR* BsmI (AA)		0.01									
*VDR* BsmI (GA_AA)				0.03							
*VDBP* GC1296 (AC_CC)									0.04		
*CYP27A1* 345 (GG)		0.02									
*CYP24A1* 3999 (CC)			0.03								
*CYP24A1* 22776 (TT)	0.05										
**B**	***p*-Values for Patients with at Least One Positive Test for SARS-CoV-2**										
*CYP27B1* −1260 (TT)			0.03								
*CYP24A1* 22776 (CT_TT)		0.02									
*VDR* ApaI (AA)							0.03	0.01			0.03
*VDR* FokI (CC)						0.04					
*CYP24A1* 8620 (AG_GG)					0.05						
*CYP24A1* 8620 (GG)						0.01					
*CYP27A1* 345 (AG_GG)						0.04					
*CYP24A1* 22776 (TT)						0.01					

**Table 3:** Median 25-vitamin D levels (ng/mL) in preterm births (N = 11) compared to full-term pregnancy (N = 107).

	TOTAL	Patients Negative for Every Test for SARS-CoV-2	Patients with at Least One Positive Test for SARS-CoV-2
25-Vitamin D trimester 1 (ng/mL)	23 (17–29)	23 (17–28)	27 (10–27)
25-Vitamin D trimester 2 (ng/mL)	25 (19–30)	24 (19–30)	28 (26–28)
25-Vitamin D trimester 3 (ng/mL)	30 (23–39)	32 (24–40)	20 (13–29)

## Data Availability

Data are available within the text. Patient data are available on request due to privacy and ethical restrictions.

## Supplementary Materials

The following supporting information can be downloaded at: https://www.mdpi.com/article/10.3390/nu14163275/s1, Table S1: The allele frequencies of the genetic variant.
