# Supplementary material for: Pregnancy and COVID-19: The Possible Contribution of Vitamin D
Source: Nutrients. 2022 Aug 10;14(16):3275. doi: 10.3390/nu14163275 (PMC9414046; doi:10.3390/nu14163275)
Supplement: Supplementary file 1 [file nutrients-14-03275-s001.zip › nutrients-1842456-supplementary.pdf]

Supplementary Materials

Table S1. The allele frequencies of the genetic variant.

POLYMORPHISM	rs	Allele 1	Heterozygous	Allele 2
VDR ApaI C>A	797523219	19.4 (CC)	50.6 (CA)	30 (AA)
VDR TaqI T>C	731236	36.9 (TT)	50 (TC)	13.1 (CC)
VDR BsmI G>A	1544410	31.9 (GG)	58.1 (GA)	10 (AA)
VDR FokI T>C	17535810	13.1 (TT)	34.4 (TC)	52.5 (CC)
VDR Cdx2 A>G	11568820	16.9 (AA)	26.3 (AG)	56.9 (GG)
VDBP GC1296 A>C	7041	30 (AA)	46.9 (AC)	23.1 (CC)
CYP27B1 -1260 G>T	10877012	55.6 (GG)	32.5 (GT)	11.9 (TT)
CYP24A1 3999 T>C	2248359	26.3 (TT)	43.8 (TC)	30 (CC)
CYP24A1 8620 A>G	2585428	27.7 (AA)	43.8 (AG)	27.5 (GG)
CYP27B1 +2838 C>T	4646536	12.5 (CC)	38.1 (CT)	49.4 (TT)
CYP27A1 345 A>G	4674345	31.9 (AA)	43.1 (AG)	25 (GG)
CYP24A1 22776 C>T	927650	26.9 (CC)	50 (CT)	23.1 (TT)
